# Tumour immunogenicity goes with the (mitochondrial electron) flow

**DOI:** 10.1002/1878-0261.13627

**Published:** 2024-03-22

**Authors:** Asma Ahmed, Stephen W. G. Tait

**Affiliations:** ^1^ School of Cancer Sciences University of Glasgow UK; ^2^ Cancer Research UK Scotland Institute Glasgow UK

**Keywords:** complex II, electron transport chain, major histocompatibility complex class I, succinate

## Abstract

Mitochondrial metabolism and electron transport chain (ETC) function are essential for tumour proliferation and metastasis. However, the impact of ETC function on cancer immunogenicity is not well understood. In a recent study, Mangalhara et al. found that inhibition of complex II leads to enhanced tumour immunogenicity, T‐cell‐mediated cytotoxicity and inhibition of tumour growth. Surprisingly, this antitumour effect is mediated by succinate accumulation affecting histone methylation. Histone methylation promotes the transcriptional upregulation of major histocompatibility complex–antigen processing and presentation (MHC‐APP) genes in a manner independent of interferon signalling. Modulating mitochondrial electron flow to enhance tumour immunogenicity provides an exciting new therapeutic avenue and may be particularly attractive for tumours with reduced expression of MHC‐APP genes or dampened interferon signalling.

## Graphical abstract

Mitochondrial electron transport chain is required for tumour proliferation and T‐cell immune responses. However, the relationship between mitochondrial metabolism, tumour growth and immunogenicity remains unclear. Mangalhara et al. show that modulating mitochondrial electron flow by inhibition of complex II induces succinate accumulation that results in increased tumour immunogenicity and cytotoxic T‐cell activation leading to reduced tumour growth.

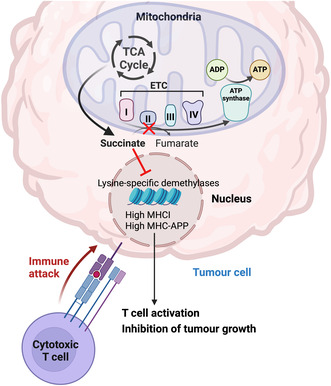

**Abbreviations**
CI: complex I
CII: complex II
ETC: electron transport chain
FADH_2_
: flavin adenine dinucleotide
KDMs: lysine‐specific demethylases
MCJ: methylation‐controlled J protein
MHC I: major histocompatibility complex class I
MHC‐APP: major histocompatibility complex–antigen processing and presentation
SDH: succinate dehydrogenase
TCA: tricarboxylic acid

Mitochondria are essential for tumour metabolism, growth and invasion. They generate cellular energy in the form of ATP through the tricarboxylic acid (TCA) cycle and the electron transport chain (ETC). The ETC is composed of four enzyme complexes; complex I (CI) that transfers electrons from NADH produced in the TCA cycle, complex II (CII), which is succinate dehydrogenase (SDH), that transfers electrons from flavin adenine dinucleotide (FADH_2_), complex III and complex IV [1, 2]. Hanahan and Weinberg [3] have highlighted the reprograming of cellular metabolism to sustain tumour proliferation and the evasion of immune destruction, as emerging cancer hallmarks. However, the interplay between metabolism and tumour immunogenicity remains unclear. Moreover, while mitochondrial ETC is key for tumour growth [2], the relative contribution of CI and CII in tumour development is poorly understood.

Upon metabolic deregulation, some metabolites can have oncogenic properties and are commonly referred to as oncometabolites. For instance, SDH mutations associated with cancers of neuroendocrine origin, lead to accumulation of succinate that can have various oncogenic functions. Succinate accumulation as a consequence of SDH mutations has been shown as an epigenetic regulator whereby succinate inhibits DNA and histone demethylases. Thus, SDH‐deficient tumours displayed a hypermethylated phenotype that is oncogenic, underscoring a potential for epigenetic approaches to cancer therapy [2, 4]. Nevertheless, despite the apparent crosstalk between metabolism and epigenetics, whether epigenetic reprogramming affects tumour immunogenicity is unclear.

Tumours often evade the immune system by downregulating the expression of major histocompatibility class I (MHC I) molecules that are key for antigen presentation and the subsequent activation of cytotoxic T cells [5]. Mangalhara et al. investigated the interaction between tumour growth, CI and CII of the ETC and tumour immunogenicity by implanting CI or CII knockout mouse melanoma cells into immunocompetent mice. The authors found that the inhibition of CII but not CI reduced tumour growth as a result of increased expression of MHC I and several major histocompatibility complex–antigen processing and presentation (MHC‐APP) genes that enhance antigen presentation and T‐cell activation potentiating T‐cell‐mediated killing of tumour cells [6] (Fig. 1). Importantly, the authors showed that the increased tumour cell antigen presentation is caused by mitochondrial succinate accumulation (resulting from reduced complex II activity) and is independent of interferon‐gamma signalling (a common means of upregulating MHC‐APP genes). The clinical relevance of these findings is underscored by an inverse correlation between the expression of SDHC (a subunit of CII) and MHC‐APP genes in both skin and breast cancer. These findings highlight the role of CII inhibition on antigen presentation and T‐cell activation and raise the question of whether CII has a role in other immune cell types.

**Fig. 1:**
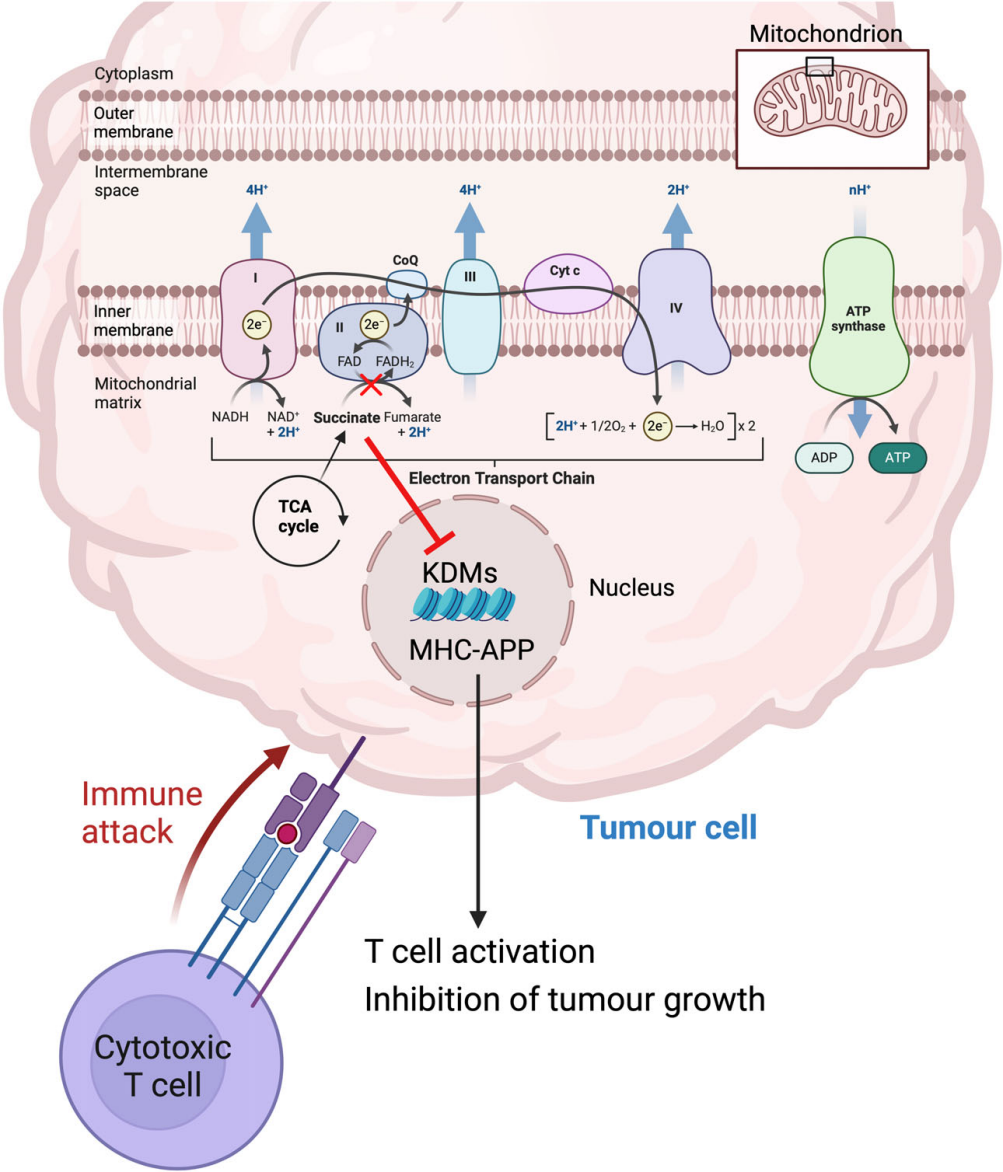
Inhibition of mitochondrial complex II (CII) induces succinate accumulation that inhibits lysine‐specific demethylases (KDMs). This causes upregulation of major histocompatibility complex–antigen processing and presentation (MHC‐APP) genes leading to increased tumour immunogenicity and cytotoxic T cell activation.

Paradoxically, loss of complex II function—causing succinate accumulation—also has reporting oncogenic functions in some human cancers, underpinning succinate as an oncometabolite [7, 8]. How can these opposing, pro and antitumourigenic, effects of succinate be reconciled? As the authors discuss, oncogenic germline mutations of CII that are present early in life, lead to succinate accumulation that promotes tumour initiation. In contrast, in the current study, succinate impacts tumour growth control by potentiating tumour immunogenicity. Intriguingly, succinate accumulation does not always lead to tumorigenesis [9] and sometimes requires additional mutations for tumorigenesis to occur [10], which indicates that the oncogenic potential of SDH loss and succinate accumulation is dependent on the microenvironment. Furthermore, CII deficiency might cause immunoediting and the selective growth of tumours that escaped immune destruction. Nonetheless, further research is needed to decipher the tumour‐promoting versus the tumour‐suppressive effects of succinate and to explore which mutations are coupled to which phenotype. Moreover, it will be important to investigate whether these antitumour effects of CII inhibition are also evident in other cancer types.

How does succinate accumulation induce MHC I expression? Metabolism and epigenetic control of gene expression are intimately linked [11]. In this vein, the authors found that the reduced α‐ketoglutarate/succinate ratio caused by the reduction in CII activity resulted in inhibition of lysine‐specific demethylases (KDMs). This led to increased trimethylation of histone H3 at lysine 4 (H3K4me3), and upregulation of the NLRC5 transcription factor and a transcriptional increase in MHC I and other APP genes expression (Fig. 1). This effect was reversed by the inhibition of histone methylation. Furthermore, H3K4me3 was significantly enriched at the promoter of Nlrc5 and Tap1. Additionally, succinate accumulation induced a marked increase of H3K4me3 in the Tap1 gene body that was rescued by α‐ketoglutarate. Thus, succinate primarily impacts MHC I expression through epigenetic effects.

The authors next sought to exploit these findings for therapeutic benefit, aiming to increase succinate levels to promote antitumour immunity. Inhibition of CII is neurotoxic and can induce inflammatory responses [12, 13] and systemic CII inhibition can induce tumourigenesis and significant disruption of the ETC in healthy cells, hence precluding direct targeting of CII as a therapeutic option. Therefore, the authors took an alternative approach to trigger succinate accumulation by targeting the ETC to enhance CI‐driven electron flow. To this end, the authors took an elegant approach, knocking out an endogenous CI‐interacting protein in the inner mitochondrial membrane, called Methylation‐controlled J protein (MCJ), that acts as a negative regulator of CI [14], on the premise that the loss of MCJ will lead to the selective activation of CI and hence the rewiring of electron flow leading to succinate accumulation and enhanced tumour immunogenicity. Indeed, MCJ knockout resulted in increased CI activity and intracellular succinate accumulation leading to immune‐mediated suppression of tumour growth.

In summary, Mangalhara et al. provide further compelling evidence that modulation of mitochondrial metabolism represents a promising therapeutic target for cancer. This can be achieved by rewiring the ETC in tumour cells without affecting normal cell respiration and thus minimising treatment side effects. Remarkably, the observed antitumorigenic effect and the enhanced immunogenicity of melanoma cells are independent of interferon‐gamma signalling. This is particularly exciting and can be utilised as a treatment strategy for tumours with defective interferon‐gamma pathways that are resistant to immunotherapy [15]. This approach could be particularly promising in immune cold tumours, by making them visible to the immune system and potentially more responsive to combinatorial immunotherapy, for example with immune checkpoint blockade. Nonetheless, the feasibility of rewiring the electron flow in a therapeutic setting requires careful consideration. Moreover, as succinate can act as an oncometabolite, it is of utmost importance to dissect the tumour‐promoting versus the tumour‐inhibitory effects of succinate before delving into enhancing succinate accumulation as a treatment strategy.

## Conflict of interest

SWGT consults for Exo Therapeutics. AA declares no conflict of interest.
